# Effects of Lower-Leg Kinesiology Taping on Balance Ability in Stroke Patients with Foot Drop

**DOI:** 10.1155/2015/125629

**Published:** 2015-10-22

**Authors:** Young-Hyeon Bae, Hyeong Geun Kim, Kyung Sam Min, Suk Min Lee

**Affiliations:** ^1^Department of Physical and Rehabilitation Medicine, Samsung Medical Center, Seoul 135-710, Republic of Korea; ^2^Department of Physical Therapy, Angelo State University, San Angelo, TX 76909, USA; ^3^Department of Physical Therapy, Hallym University, Seoul 200-702, Republic of Korea; ^4^Department of Bioengineering, University of Pittsburgh, Pittsburgh, PA 15260, USA; ^5^Department of Physical Therapy, Sahmyook University, Seoul 01795, Republic of Korea

## Abstract

*Objective*. The purpose of this study was to observe the effects of lower-leg kinesiology taping on balance ability in stroke patients with foot drop. *Design*. Randomized controlled trial study. *Method*. Thirty stroke patients with foot drop were randomly divided into two groups. The experimental group underwent kinesiology taping, and the control group underwent placebo taping. Balance ability was assessed before and after taping in both groups. *Results*. No difference was observed over time in the Berg Balance Scale score between the two groups, and a significant difference in the Berg Balance Scale score was observed only in the experimental group. Additionally, there were significant differences in the center of pressure area and limits of stability over time. *Conclusion*. Kinesiology taping temporarily improved static balance ability in stroke patients. However, its effect on dynamic balance was not verified. Therefore, further research on the influence of long-term kinesiology taping on dynamic balance and gait ability is suggested.

## 1. Introduction

Approximately 61–80% of the body weight in stroke patients is shifted to the unaffected lower extremity; this leads to an asymmetrical standing position [1, 2]. This position causes a decline in balance ability as the body's center of mass is shifted to the unaffected side, leading to a disruption of symmetrical weight shifting in response to external movement [3]. Decline in balance ability, besides increasing lower extremity stiffness, also disrupts independent walking, thereby exacerbating the sense of chronic disability [1, 2]. Moreover, 84% of stroke patients have involvement of more than one joint, and in 76% of these, the ankle joint is involved [4]. Weak muscles and instability of the ankle joint cause a foot drop in stroke patients [1, 2]. Thus, problems with balance lead to increased difficulties in recovery of activities of daily living (ADL) function and motor function and increase the possibility of injury from a fall [5]. A decline in balance ability due to an asymmetrical weight load adversely affects general body movement as it leads to unavoidable compensatory strategies involving the unaffected side.

Strengthening of ankle muscles can improve range of motion (ROM) and ADL performance [6]. To improve abnormal walking caused by instability of joints of the foot, a combination of both medical and nonmedical therapies is required as medical intervention alone is insufficient [1, 2]. Additionally, the ankle joint not only supports the body through cooperation of the weight loading process and lower limb muscle function, but also provides sensory information and repetitively stimulates the sense of postural maintenance through the touch of the sole of foot on the ground during movement [7]. In regard to balance and gait ability, the ankle plays a sponge-like role and controls the first stage of balance due to posture disturbance. Therefore, muscle strengthening and improved ROM of the ankle are required to improve body control and walking ability [1, 2, 8, 9].

The ankle foot orthosis (AFO) is used commonly in the clinical setting to treat a foot drop; however, it has several disadvantages such as discomfort, fixation of the ankle in immobile positions as in sitting, weakening of muscles around the ankle joint as it is maintained in a fixed position during gait, and lack of availability of appropriate shoe sizes that allow inclusion of the AFO [10]. The AFO has been shown to have a positive effect on balance, gait speed, and gait stride in patients with a recent stroke, but it did not show a positive effect with increase in the duration since onset [10]. Kinesiology taping (KT) of the ankle increases stimulation and support depending on the application method [11, 12]. Moreover, it is reported that KT improves gait and balance by allowing a stronger transfer of signal of skin receptor, increasing proprioception, and enabling better joint alignment, which results in improved joint stability [13]. KT for improved balance and gait is usually applied to the ankle to improve feedback of proprioceptive and recruitment of the stabilizing muscles [1, 2, 8, 9]. Taken together, KT improves balance and gait ability by increasing the proprioceptive sense input and enabling accurate joint position sense of the ankle [14]. This previous study showed that Mulligan ankle KT did not have a significant response on postural control [15]. But KT of gastrocnemius muscle does significantly respond to the lateral gastrocnemius muscle activity [16]. And KT of ankle to the paralyzed side of a stroke patient has improved on typical asymmetric gait and gait speed [8].

Therefore, the purpose of this study was to observe the effects of lower-leg KT of paralyzed side on balance ability in stroke patients with foot drop.

## 2. Methods

### 2.1. Subjects

In this study, 30 stroke patients with foot drop, who met the below-mentioned criteria and agreed to participate, were selected randomly. The inclusion criteria were as follows: (1) occurrence of a stroke in the past 3 months based on computed tomography or magnetic resonance imaging; (2) dragging of toes when walking due to the foot drop; (3) Modified Ashworth Scale of the ankle with Modified Ashworth Scale grade <2; (4) use of a plastic AFO; (5) ability to maintain standing position and walk at least 10 m independently. Exclusion criteria applied during screening included the following: (1) cognitive deficit; (2) fracture or nonspecific skin disease; (3) any history of pain; (4) ankle surgery; (5) vertigo, dizziness, and/or any balance-related disorder [15, 16]. Power calculations indicated that a sample of more than 30 subjects would provide an 80% (*β* = .20) chance of detection of a 20% (*α* = .05) difference in improvement between two groups. The patients were randomly divided into the experimental (*n* = 15) and control (*n* = 15) groups (Table 1).

### 2.2. Procedures

Subjects did not wear an orthosis during the experiment. The balance ability of the subjects was assessed primarily with intervention. Lower-leg KT was applied to the subjects in the experimental group and placebo taping was applied to those in the control group. Balance ability was assessed based on the Berg Balance Scale (BBS) score, the Gaitview system, which assessed the center of pressure area (COPA), and the BioRescue, which measured the limits of stability (LOS).

### 2.3. Taping Intervention

**Figure 1:**
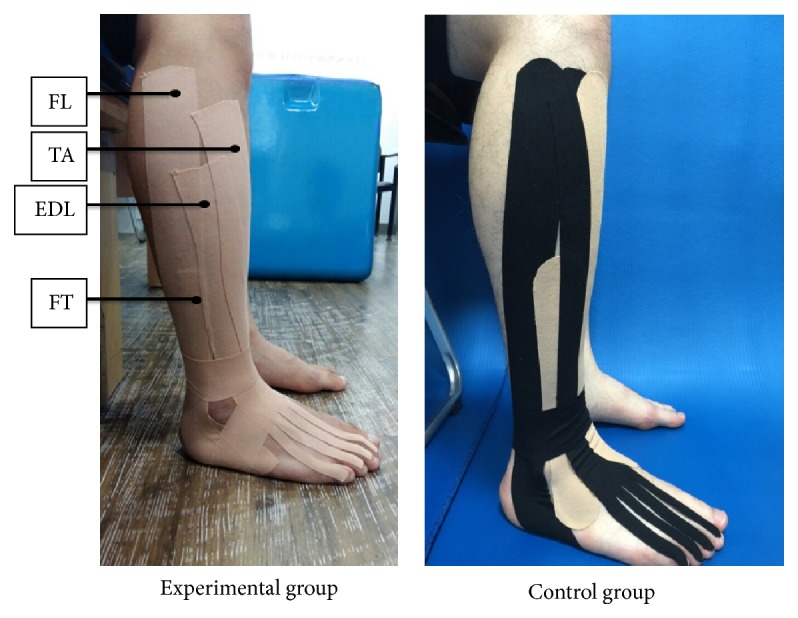
Kinesiology taping technique. Experimental group is using the kinesiology tape on the muscles and control group is using the inflexible tape on the same muscles. FL: fibularis longus muscle; FT: fibularis tertius; EDL: extensor digitorum longus; TA: tibialis anterior.

The flexible kinesiology tape was attached on the skin of the experimental group subjects without elongation, after maximal extension of the muscles. KT were applied using the kinesiology tape (BB Tape, WETAPE Inc., Seoul, Korea) by physical therapist who has KT certification with more than 10 years of experience. The tape was attached on the fibularis longus, fibularis tertius, extensor digitorum longus, and tibialis anterior, and additional support taping was applied around the ankle. The KT for these muscles was applied from the foot to the knee on the affected side, along the direction of the muscle fibers with the ankle in plantar flexion (Figure 1). In the control group, an inflexible tape was attached only on the front of the lower leg, in the same manner as the kinesiology tape, for a placebo effect. No additional ankle support taping was applied [15–17].

### 2.4. Measurements

#### 2.4.1. BBS

The 14-item BBS identifies and evaluates balance impairment in patients with hemiplegia and has been reported to be responsive to clinically meaningful changes. When a subject was unable to independently complete a test item, he/she was given 3 attempts, and the score on the best attempt was recorded. A total score for all items was determined for each subject (maximum score = 56 points) [5, 8].

#### 2.4.2. COPA

The Gaitview system, AFA-50 (Alfoots Co., Korea), marks the movement of the center of body pressure on the monitor, quantitatively measures that area, and provides quantitative information related to postural stability and weight shifting. Balance ability was assessed based on the symmetry of plantar pressure in the standing position. The subject was required to go up on a pad barefoot and maintain the standing position. Following this, the subject had to maintain his balance for 30 seconds, while the distance traveled by the center of pressure was measured. Each subject was evaluated twice in the same manner, and the average was calculated for each measurement [18].

#### 2.4.3. LOS

The BioRescue system (biofeedback AP1153, BioRescue Co., France) is composed of a depression platform that can measure power in various ways and a motion analysis system made by RMI Inc. The system measures the pathway of center of pressure during a specific movement and consequently the area of the pathway. The experimental method was demonstrated prior to the experiment, during which the subject was instructed to maintain an erect posture in standing with 30-degree wide base between the feet. While the subject maintained his balance for 30 seconds with open eyes, the total movement area (mm^2^) of the body's center of pressure was measured to evaluate his static stability. Using the same method, the evaluation was performed twice, and the average value was calculated [19].

### 2.5. Data Analysis

The measured data were analyzed using SPSS, version 21.0. Using the Kolmogorov-Smirnov test, we determined that all the parameters were normally distributed. Additionally, for a comparison of the general characteristics of the subjects, the chi-square test and independent *t*-test were performed. Each group had a homogeneous dependent variable (*p* > 0.05). Finally, the paired *t*-test was performed for each group to compare the changes before and after the intervention. The two-way repeated measures ANOVA was also performed to compare the changes over time between the two groups. The statistical significance level was set as 0.05.

## 3. Results

### 3.1. Changes in the BBS Score

The BBS score of the experimental group changed significantly from 41.91 ± 7.05 to 44.66 ± 6.99 after the intervention (*p* < 0.05). The BBS score of the control group changed significantly from 43.25 ± 5.91 to 44.00 ± 5.18 (*p* < 0.05). There was no significant difference over time between the two groups (Table 2).

### 3.2. Changes in the COPA and LOS

The COPA and LOS of the experimental group changed significantly from 4.58 ± 2.44 cm^2^ to 4.04 ± 2.26 cm^2^ and from 9906.50 ± 1750.97 mm^2^ to 10922067 ± 1827.383 mm^2^ (*p* < 0.05), respectively. The COPA and LOS of the control group changed significantly from 5.24 ± 2.67 cm^2^ to 5.19 ± 2.55 cm^2^ and from 9915.25 ± 1856.99 mm^2^ to 9966.75 ± 1854.28 mm^2^ (*p* < 0.05), respectively. The COPA and LOS over time were significantly different between the two groups (*p* < 0.05) (Table 2).

## 4. Discussion

The AFO is commonly used in stroke patients with foot drop. It is easily detachable and washable, light, and well-suited for use with various shoes [20–22]. However, a fixed orthosis disturbs the normal kinematics of the ankle joint and impedes sensory feedback during gait. Bulley et al. (2011) reported that the use of the AFO results in a fixed ankle, which leads to an abnormal gait pattern and weakening of surrounding muscles; therefore, the use of an AFO does not provide long-term relief from foot drop symptoms [10]. According to Karlsson and Andreasson (1992), improved ankle movement control and contraction of the ankle stabilizers were observed after application of kinesiology tape to the ankle [23]. In addition, the hip and ankle muscles in a normal elderly group have been shown to be significantly different from those in a group of individuals with an insecure gait and at an increased risk for falls [24, 25]. Therefore, KT is used widely to revitalize the ankle strategy for balance. The mechanism of KT can be explained by gamma motor reflexes of the skin. Adhesion of the kinesiology tape on the skin allows continuous muscle contraction and induces relaxation of muscle tone by inputting information about the level of muscular contraction and allowing repetitive muscle contraction and release [1, 2, 8, 9]. KT is applied along the grain of the muscle, which allows joint movement, increases the space between muscle and skin and consequently increases the blood and lymph circulation, and improves motor function [1, 2, 8, 9, 26]. Thus, the effects of lower-leg KT in stroke patients with foot drop were identified, and it was suggested as an ancillary method in these patients.

The major functions of the foot and ankle are to provide momentum during gait, shock absorption, and balance control. Therefore, for the improvement of these functions, ankle ROM and muscle strength and proprioception are important [5]. Specifically, the anterior tibialis and medial calf muscles are activated in response to posture disturbance. The calf muscles are activated by forward movement, and the anterior tibialis muscle is activated by backward movement [27]. A foot drop, seen in stroke patients, is a result of stiffness and paralysis, which causes instability and weakness of the foot and ankle and negatively affects gait [28]. Specifically, the ankle joint works to maintain balance when the posture disturbance is minimum. This ankle strategy is accomplished by alternative activation of the tibial and medial sural muscles. Additionally, the medial sural muscle is activated when the body moves forward, and the tibial muscle is activated when the body moves backward [27]. Stroke patients, therefore, present with disturbance of balance ability and postural control [29]. For this purpose, the kinesiology tape was applied to the peroneus longus, extensor digitorum longus, and anterior tibial muscle of the lower leg, while support taping was applied around the ankle.

In this study, there was no difference in the BBS score between the two groups. In two previous studies, which reported the correlation of gait ability with balance and sensory function [5] and a significant correlation between balance abilities in walking and standing [30], limited effects of temporary KT on improvement of stationary and kinetic balance were noted. However, the COPA and LOS of the experimental group significantly decreased over time, which resulted in improvement of stationary balance ability in our study. Callaghan et al. (2002) reported improvement in the joint range and cognition with respect to position sense [31]. Gross et al. (1997) also reported the effectiveness of KT on improvement of lower-leg function [32]. These observations were in agreement with the results of a previous study, which reported that KT restricts excessive movement of the joint and improves proprioceptive feedback, consequently shortening the mobilization time of the stabilizing muscles of the joint [23].

## 5. Conclusion

In conclusion, temporary KT positively affects stationary balance in stroke patients. However, its effects on kinetic balance were not verified. The limitation of this study is that long-term effects of KT were not investigated, as the tests were conducted immediately after applying the tape. Further investigation of the effects of long-term KT application on kinetic balance in stroke patients with foot drop is needed.

## Figures and Tables

**Table 1:** General characteristics of the subjects.

	Kinesiology taping group	Placebo group	*t*
Age (years)	65.08 ± 9.33	63.50 ± 5.90	1.311^*∗*^
Height (cm)	164.08 ± 6.93	161.33 ± 8.46	0.640^*∗*^
Weight (kg)	68.85 ± 8.90	58.83 ± 8.63	0.939^*∗*^

Note: values are expressed as means ± standard deviation (SD). *∗* indicates no significant difference.

**Table 2:** Comparison of balance ability changes between two groups.

		Kinesiology taping group	Placebo group	*F*	*p*
BBS (score)	Before	41.91 ± 7.05	43.25 ± 5.91		
	During	44.66 ± 6.99	44.00 ± 5.18	2.066	.165
	*p*	.004	.482		

COPA (cm^2^)	Before	4.58 ± 2.44	5.24 ± 2.67		
	During	4.04 ± 2.26	5.19 ± 2.55	15.803	.001
	*p*	.001	.545		

LOS (mm^2^)	Before	9906.50 ± 1750.97	9915.25 ± 1856.99		
	During	10922.67 ± 1827.383	9966.75 ± 1854.28	22.135	.000
	*p*	.000	.744		

Note: values are expressed as means ± standard deviation (SD).
